# A cluster-randomised clinical trial comparing two cardiovascular health education strategies in a child population: the Savinghearts project

**DOI:** 10.1186/1471-2458-12-1024

**Published:** 2012-11-23

**Authors:** Luis María Sánchez-Gómez, María Jesús Fernández-Luque, Lourdes Ruiz-Díaz, Rosa Sánchez-Alcalde, Belén Sierra-García, Soledad Mayayo-Vicente, Marta Ruiz-López, Pilar Loeches-Belinchón, Javier López-Gónzález, Amelia González-Gamarra, Angela Gallego-Arenas, Ana Cubillo-Serna, Gema Gil-Juberias, Pilar Pérez-Cayuela, Celina Arana Cañedo-Arguelles, Julia Natividad García-Pascual, Esther Ruiz-Chércoles, Carmen Suarez-Fernández, Iluminada Garcia-Polo, Daniel Abad-Perez, Juan M Ballesteros-Arribas, Maravillas Izquierdo-Martínez, Elena Salvador-Alcaide, Ana B Arribas-Vela, Juan M Alonso-Pérez, Lorena Veja-Piris, Francisco Rodríguez-Salvanés, Blanca Novella-Arribas

**Affiliations:** 1 Agencia de Evaluación de Tecnología Sanitarias (AETS), ISCIII. Instituto de Investigación Sanitaria del Hospital Universitario de La Princesa (IP). C/ Monforte de Lemos 5, Madrid, 28029, Spain; 2 Área de Formación Especializada, Agencia Laín Entralgo, Consejería de Sanidad, Comunidad de Madrid, Instituto de Investigación Sanitaria del Hospital Universitario de La Princesa (IP), C/ Gran Vía 27, Madrid, 28013, Spain; 3 D.G. de Atención Primaria, SERMAS Consejería de Sanidad, Comunidad de Madrid, Instituto de Investigación Sanitaria del Hospital Universitario de La Princesa (IP). Red Temática de Investigación en Enfermedades Cardiovasculares (RECAVA), Pza. Carlos Trías Bertrán, 7, Madrid, 28020, Spain; 4 FREMAP, Instituto de Investigación sanitaria del Hospital Universitario de La Princesa (IP), Avenida Pablo Iglesias 36-40, Madrid, 28039, Spain; 5 D. G. de Gestión Económica y de Compras de Productos Sanitarios y Farmacéuticos, Consejería de Sanidad, Comunidad de Madrid, Pza. Carlos Trías Bertrán 7, Madrid, 28020, Spain; 6 Servicio de Medicina Interna, Hospital Universitario de la Princesa. Instituto de Investigación sanitaria del Hospital Universitario de La Princesa (IP). Red Temática de Investigación en Enfermedades Cardiovasculares (RECAVA), (C/ Diego de León 62), Madrid, (28006), Spain; 7 Ministerio de Sanidad, Política Social e Igualdad, (Paseo del Prado 18-20), Madrid, (28014), Spain; 8 Subdirección de Promoción de la Salud y Prevención, D.G. de Atención Primaria Madrid, SERMAS, Consejería de Sanidad, Comunidad de Madrid, (C/ Julián Camarillo, 4B), Madrid, (28037), Spain; 9 Consejería de Educación y Empleo, Comunidad de Madrid, (C/ Alcalá, 30 – 32), Madrid, (28021), Spain; 10 Unidad de Información Clínico Asistencial. Servicio de Admisión y Documentación Clínica, Hospital Universitario de la Princesa. Instituto de Investigación sanitaria del Hospital Universitario de La Princesa (IP). Red Temática de Investigación en Enfermedades Cardiovasculares (RECAVA), (C/ Diego de León 62), Madrid, 28006, Spain

**Keywords:** Cluster randomised clinical trial, Cluster analysis, Cardiovascular diseases, Child obesity, Educational concerts, Healthy strategies, NAOS strategy

## Abstract

**Background:**

This paper describes a methodology for comparing the effects of an eduentertainment strategy involving a music concert, and a participatory class experience involving the description and making of a healthy breakfast, as educational vehicles for delivering obesity-preventing/cardiovascular health messages to children aged 7–8 years.

**Methods/design:**

This study will involve a cluster-randomised trial with blinded assessment. The study subjects will be children aged 7–8 years of both sexes attending public primary schools in the Madrid Region. The participating schools (n=30) will be randomly assigned to one of two groups: 1) Group MC, in which the children will attend a music concert that delivers obesity-preventing/cardiovascular health messages, or 2) Group HB, in which the children will attend a participatory class providing the same information but involving the description and making of a healthy breakfast. The main outcome measured will be the increase in the number of correct answers scored on a knowledge questionnaire and in an attitudes test administered before and after the above interventions*.* The secondary outcome recorded will be the reduction in BMI percentile among children deemed overweight/obese prior to the interventions. The required sample size (number of children) was calculated for a comparison of proportions with an α of 0.05 and a β of 0.20, assuming that the Group MC subjects would show values for the measured variables at least 10% higher than those recorded for the subjects of Group HB. Corrections were made for the design effect and assuming a loss to follow-up of 10%. The maximum sample size required will be 2107 children. Data will be analysed using summary measurements for each cluster, both for making estimates and for hypothesis testing. All analyses will be made on an intention-to-treat basis.

**Discussion:**

The intervention providing the best results could be recommended as part of health education for young schoolchildren.

**Trial registration:**

Clinicaltrials.gov: NCT01418872

## Background

Cardiovascular disease is the leading cause of death in the Western world [1]; indeed, in 2008 it was the cause of 31.8% of all deaths registered. In Spain, in that same year, ischaemic heart disease (IHD) was the most common cause of death among men (20,369 fatalities), while in women, stroke was the major cause of demise (18,312 deaths). The second cause of death among women was, again, IHD (15,519 fatalities) [2]. Numerous epidemiological studies have shown associations between different cardiovascular risk factors (CRF) and the appearance of these diseases and several of the classic CRFs (diabetes, obesity, high blood pressure and high cholesterol) are known to be influenced by poor dietary habits and the undertaking of little physical activity. These habits are hard to modify in adult life; it is therefore important to improve the development of lasting healthy habits during childhood [3,4].

Obesity is a CRF that has increased alarmingly in importance in recent years; indeed, the world is now faced with an obesity pandemic [5]. The World Health Organization (WHO) estimates there to be 1000 million overweight people worldwide, of which 300 million are obese [6]. Although the appearance of obesity is complex and influenced by genetic and other biological factors, poor diet and the undertaking of little physical inactivity are also involved. According to the National Health and Nutrition Examination Survey (NHANES) [7], obesity affects 17% of children and adolescents in the US. In fact, over the last 30 years the prevalence of obesity has nearly tripled in Australia, Brazil, Canada, the USA, Germany, France, Greece, the UK, and Japan (although it would now appear to be stabilizing in Sweden, the USA and the UK). In 2010, the prevalence of overweight/obesity was estimated at 40% for North America and the countries of the Eastern Mediterranean, at 38% for Europe, at 27% for the Western Pacific, and at 22% for Southeast Asia [8]. It is now known that overweight and obesity in childhood are associated with an increased risk of hypertension, diabetes, dyslipidemia and obesity in adulthood [9,10]. A number of studies from the USA (including the Bogalusa Heart Study) [11,12] and Finland [13,14] have shown that diseases that appear during older age may have their origin in earlier stages of life.

In Spain, the EnKid study showed the prevalence of overweight/obesity to be 30.4% among children aged 6–9 years (overweight alone = 14.5%, obesity alone = 15.9%) [15], while the Spanish National Health Survey showed 25.7% of girls and 29.3% of boys (aged 2–17 years) to be overweight/obese [16]. The International Obesity Taskforce (IOT) experts group reported the prevalence of childhood obesity in Spain to be among the highest in Europe [17], and in 2009, the Spanish system for the surveillance of risk factors associated with non-communicable diseases (SIVFRENT) highlighted the need to prioritise the surveillance of indicators related to energy balance given the increase observed in the prevalence of overweight/obesity, especially in girls [18]. Obesity, sedentary lifestyles, and poor nutritional and dietary habits are all increasing among the child population [19]. This age group should therefore be considered a priority for interventions aimed at promoting healthier lifestyles [20].

A review by the Cochrane Collaboration [21], in which 22 studies on improving either dietary habits or physical activity in children were analysed, concluded there to be insufficient data to affirm that the interventions undertaken helped prevent obesity. However, when changes were made to both the diet *and* physical activity, a small but positive effect on body mass index (BMI) was confirmed. However, other authors [22], who examined 158 studies mostly conducted in schools, emphasized the importance of exercise in achieving weight loss and preventing the appearance of chronic diseases in later life (note that some of these studies were reported to have problems in terms of their methodological quality).

In Spain, the Catalan Agency for Technology and Medical Research (AATRM) recently published a clinical practice guide for the prevention and treatment of childhood obesity [23]. This guide offers 64 evidence-based recommendations, 37 of which include the promotion of healthy eating habits and physical activity via educational programs that involve families and institutions. Spanish interventions aimed at preventing obesity take their inspiration in the home-grown PERSEO programme and the international NAOS strategy (Strategy for Nutrition, Physical Activity and Obesity Prevention) [24]. Both encourage information campaigns and agreements between public and private institutions in the health sector and beyond that could help goals be attained.

Townsend et al. reported that children make better food choices if schools promote healthy eating [25], and many studies have shown that inadequate breakfasts cannot be compensated for by other meals during the day [26-31].

One of the obesity prevention programmes promoted in schools is the “Healthy Breakfast Programme” [32]. This involves teaching primary schoolchildren the importance of having a good breakfast and of undertaking exercise. Public health workers, nurses and doctors at nearby health centres come to schools to teach – in the school dining room - that an adequate breakfast is important for all age groups, but especially for children. The Healthy Breakfast programme promotes 1) a breakfast consisting of a glass of full fat milk, two slice of bread with virgin olive oil, and a fruit (all key elements of the Mediterranean diet and representative of four food groups), and 2) physical activity through games in the playground. Primary school pupils usually aged 7–8 years receive a class on the importance of these points (see *Intervention Groups, Healthy Breakfast Group* below for a more detailed description), and take a reinforcing information pamphlet home. The programme has reached 37000 children over its 14 years of existence and has improved eating habits, e.g., the percentage of schoolchildren who have breakfast everyday has risen from 88% to 91.5% over this period [30]. Further, among those who already took breakfast, the composition of this meal has improved, with 84.4% now including four or more foods in this meal compared to a prior 77.4%. The motto of the campaign is “*First eat your breakfast, and then eat your day*” [32].

Music interventions have often been used to promote learning, communication and even to attain clinical goals [33-36]. In recent years, music has been used as part of cardiovascular health education. Williams et al. [37] showed that education via hip-hop music, used as a medium to deliver health messages to teenagers regarding strokes, reduced the time for assistance to be sought for adult stroke victims. In 2001, Marconato et al. [38] showed that including classical music in health education sessions reduced anxiety levels in students aged >18 years, increased personal happiness, promoted the consumption of fibre-rich foods, and reduced the intake of high cholesterol foods. Thus, music could be used to deliver educational messages in interventions aimed at promoting obesity prevention and the adoption of good cardiovascular health habits. In the present work, this music will be offered as an eduentertainment package [39], and will be provided by specialist musicians who have been involved in this type of project for some three years in the Madrid Region [40,41].

The proposed project aims to compare the effectiveness – in terms of knowledge and attitudes regarding obesity prevention/good cardiovascular health practices – of a health promotion strategy involving an Healthy Breakfast with that of an educational concert that delivers the same health messages via the vehicle of music and storytelling.

### Aims

The main aim of the proposed trial is to determine whether a health promotion strategy involving an educational music concert for schoolchildren aged 7–8 years improves knowledge of, and attitudes towards, obesity prevention/good cardiovascular health habits (as suggested by the NAOS strategy) better than a health promotion strategy involving an Healthy Breakfast.

The secondary aim is to compare the two interventions, at 6 months, in terms of the reduction achieved in BMI percentile by children who are overweight/obese prior to the start of the study.

## Methods

This study (ClinicalTrials.gov Identifier Number: NCT01418872) is a cluster-randomised clinical trial with parallel groups. A cluster design was chosen given the interest in performing the project in schools, and to prevent the contamination problems that would be faced if the children were the units of randomisation.

### Study population

Thirty public primary schools (clusters) will be selected among those that opt to be included in the proposed project (known as the Savinghearts Project) as one of the elective course offered by the Madrid Region education system. Thirty centres with at least two 2^nd^ grade classes (children’s ages 7–8 years) will be chosen by random sampling, stratified by rural/urban location and socioeconomic level. Each of the schools selected will be assigned a number and then randomised (using a random numbers table) to one of the intervention groups below (see *Intervention Groups*).

#### Inclusion criteria

Children must be present on the day of the planned activities, their parents or guardians must have given their informed consent for them to be included, and they should be subject to no exclusion criteria.

#### Exclusion criteria

Children with food allergies or food intolerances, with uncorrected severe hearing impairment, whose parents do not give their informed consent, with a poor understanding of oral and written language, or who have previously been involved in the Healthy Breakfast Programme, will be excluded.

#### Loss to follow-up

Withdrawals will be recorded when parents or tutors rescind consent for a pupil to continue. Few withdrawals may be expected since both proposed interventions are one-off experiences and are associated with no adverse effects. Further, the duration of the trial is only 6 months. Those children who do not complete the required questionnaires 7 days and 6 months after the interventions will also be considered lost to follow-up. Such problems can be minimised since the collaboration of the participating centres’ staff can be counted upon; indeed, the class teachers will be charged with passing out these questionnaires. Children will be weighed and measured on the day of the interventions and 6 months later. However, the nurse hired for the project will also go to the schools 3 days after the second measurement to measure those children who were not present, thus reducing loss to follow-up.

### Intervention groups

•Healthy Breakfast group (Group HB)

This group will receive a Healthy Breakfast class. The activity will be held in school hours in the school dining hall, and will take 1 h to complete. A maximum of 50 children per turn will be admitted. The session will be structured as follows:

• *Preparation of the dining hall for the activity (10 min).* This involves putting out a table mat, a cup, a plate and two slices of bread per child, a 1 L bottle of milk for every four children, and a 1 L bottle of virgin olive oil for every 12 children. The dining hall will be set up before the children come in.

•Introduction to the speakers by a teacher (5 min).

• *Participatory lesson (20 min)*. This will be supported by a Power Point presentation based on the NAOS strategy with the following basic contents: the ideal number of daily meals, the importance of breakfast for the day’s activity, leaving sufficient time for breakfast, the basic components of a healthy breakfast, the role of cereals at breakfast, the role of dairy products at breakfast, the role of fruits at breakfast, the importance of exercise for cardiovascular health, and the outcome of unhealthy habits (sedentary lifestyle).

• *Healthy breakfast time (25 min)*. The children will consume the milk, the piece of fruit and the slices of bread with oil.

#### Farewell and closing

The children will be invited to take home the table mat and the cup they used; these will be inscribed with the slogan: “I am a Heartsaver”. This will serve as a reminder of the activity. A pamphlet on the NAOS strategy will also be provided for them to take home.

•Music concert group (Group MC)

The members of Group MC will attend an educational, participatory, music concert that delivers the same health messages imparted to Group HB, but through the vehicle of music and storytelling. The concert will take place within school hours in the school auditorium or gym over a period of 1 h. A maximum of 50 children will be present per turn. The session will be structured as follows.

• *Preparation of the auditorium/gymnasium for the activity (10 min).* This involves putting out the chairs with a concert programme on each, the arrangement of music stands for four musicians and a storyteller on the stage, and setting up a NAOS strategy Power Point presentation used to project reinforcing images (e.g., children eating healthily, running or playing etc.). All these will be arranged before the children come in.

•Introduction of the musicians by the storyteller (5 min).

• *Beginning of the concert*. The concert, which involves five songs and a story based on the NAOS strategy goals (outlined in Appendix 1)*,* lasts 40 min. Children will be asked to clap, raise their hands at appropriate moments, and to become ‘Heartsavers’.

• *Farewell and closing*. The children will be invited to take the concert programme home as a bookmark, along with a cup, both inscribed with: “I am a Heartsaver”. These should serve as reminders of the activity. A pamphlet on the NAOS strategy will also be provided for them to take home.

### Study stages and variables measured

The variables measured will be:

• *Demographic variables*. Age, date of birth and gender information will be collected from all participating children.

• *Knowledge variables*. The children’s knowledge of obesity-prevention/good cardiovascular health practices will be measured via a true/false multiple choice questionnaire composed of 10 questions adapted from the 10 messages of the NAOS strategy. These questions were designed by educators and teachers of the Healthy Breakfast Programme. This questionnaire will be the same for the children in both Groups HB and MC, and will be answered in the classroom the day before the interventions, and then again 7 days and 6 months later. The outcome variable measured will be the number of correct answers.

• *Attitude variables*. These will be measured using a set of 10 cards, each with different exercises, in which students must chose the healthy attitudes from the variety offered. This activity, to be undertaken by all children in the study, will be performed during regular school time 5 school days prior to the interventions and then 6 months after the interventions. The outcome variable recorded will be the number of correct answers.

• *Weight change*. Reductions in obesity will be recorded in both Group MC and HB. Weight and height measurement will be measured, using a single, calibrated portable scale and a portable stadiometer, by a single trained nurse hired for the project. All children will be so measured on the day of the interventions and again 6 months later. The outcome variable recorded will be the percentage of children who reduced their BMI percentile out of those children considered overweight/obese prior to the interventions.

### Assessment of efficacy

The efficacy of the different strategies will be measured using the following variables.

**Figure 1:**
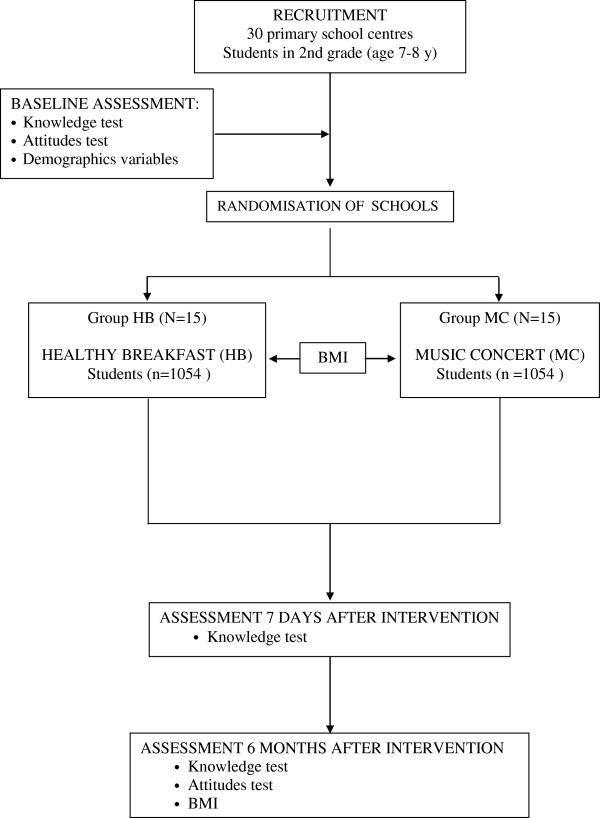
Study design and participant flow through the trial.

• *Recent knowledge acquisition*. Difference in the percentage of correct answers recorded for the first knowledge questionnaire (prior to the interventions) and the second knowledge questionnaire (7 days later) (Figure 1). Marking will be performed by two trained teachers (members of the research team), both of whom will be blinded to the groups to which the children belonged.

• *Memory of the knowledge acquired*. Difference in the percentage of correct answers between the first knowledge questionnaire (prior to the interventions) and the third knowledge questionnaire 6 months later (Figure 1). Marking will be performed by the same two teachers, both of whom will be blinded to the groups to which the children belonged.

• *Change in attitudes*. Difference in the percentage of ‘right attitudes’ recorded in the attitude test conducted 7 days before the interventions and 6 months after (Figure 1). The assessment of these attitudes will be undertaken by the same two teachers, both of whom will be blinded to the groups to which the children belonged.

• *Reduction in obesity*. Change in BMI percentiles of children originally classified as overweight/obese. The evaluation will be performed by a paediatrician (a project team member), who will be blinded to the groups to which the children belonged.

### Assessment of safety

There are no safety problems foreseen for this trial. Both strategies are considered free of adverse reactions.

### Sample size

The units of randomisation in this work are not the children, but their schools. Thirty schools will be included in the study. The necessary sample size (i.e., number of children) is determined assuming that the MC intervention can be deemed effective if it achieves a 10% improvement in knowledge of obesity prevention/good cardiovascular health practices (as outlined in the NAOS strategy) over that achieved by the HB intervention (assumed to achieve a 40% improvement over a no intervention scenario).

If simple random sampling (i.e., of the children) were used, the sample size necessary to reject the null hypothesis could be calculated in the usual way [42] for comparing two proportions - in this case involving an assumed 40% improvement with the HB intervention and the minimum 50% requried for the MC intervention to be deemed an improvement). For an α of 0.05 and a β of 0.20, the sample size required would be: n=391 × 2=782.

However, the use of a randomised cluster design with a fixed number of clusters requires the sample size be increased. According to Campbell [43], the design effect (DE) can be calculated as: DE=1 + (ñ – 1) × r, where ñ is the mean cluster population size and r the intracluster correlation coefficient (ICC). If n=782, and the study is conducted in 30 schools, a mean cluster population size (ñ) of 782/30=26 can be assumed. The sample size can then be calculated by estimating an ICC that could vary from 0.01 to 0.05. For an ICC of 0.01 the DE would be 1 + 1 × 0.01 = 1.3, and for an ICC of 0.05 the DE would be 1 + 1 × 0.05 = 2.5. Given these estimated DE values, the sample size would vary from 1009 to 1916 subjects. Assuming a loss to follow-up of 10%, the final sample size would vary between 1110 and 2107 subjects.

EPIDAT 3.1 software was used to calculate the sample size.

### Statistical analysis

Primary analysis will be made on an intention-to-treat basis. The stages of the analysis will be:

1. *Descriptive analysis*. Quantitative variables will be described by their measures of central tendency, i.e., the mean or median (in the case of asymmetric distributions) plus the respective standard deviation or interquartile range. Qualitative variables will be described in terms of ratios.

2. *Comparison of the baseline characteristics of both groups*. The characteristics of the children in both trial arms, and of their families and their schools, will be described and compared. Special attention will be paid to variables that may influence the results. Parametric tests will be used to verify the effectiveness of randomisation. Potential confounders will be identified and appropriate adjustments made in the analysis of the primary efficacy variables.

3. *Primary efficacy analysis*. Relative risk, absolute risk reduction, and the relative reduction of risk will be used as measures of the strength of associations. The null hypothesis is that there are no differences between the two interventions in terms of improving the knowledge and skills associated with obesity prevention/good cardiovascular health habits. Multivariate analyses will be performed taking into account the type of design employed via the use of generalized linear models or multilevel models. The 95% confidence interval will be calculated for all estimates. A sensitivity analysis will be performed, assigning values supportive of the null hypothesis to all missing data.

All analyses will be made using PAWS and/or STATA software.

## Discussion

This study compares the effects of an eduentertainment strategy involving a music concert, and a participatory class experience involving the description and making of a healthy breakfast, as educational vehicles for delivering obesity-preventing/cardiovascular health messages to children aged 7–8 years. The high prevalence of obesity in children and adolescents [44], and the influence this has on cardiovascular risk factors in adulthood [42], require that educational strategies to raise awareness of cardiovascular health-promoting lifestyles among children be tested.

The project suggests the developments of an innovative formula at the school context, pursuing a double objective: to explore the effect of a communication strategy based on eduentertainment, considering music as a cultural vehicle and empowering children and educators in acquiring self-care and healthy lifestyle habits.

Randomisation by clusters will be followed in the proposed work rather than simple randomisation (i.e., of the children), despite the lower statistical power of this method [45]. This has the advantage that, since all the 7–8 year-olds at each centre will be involved, it will avoid the contamination that might occur between them if they themselves were the units of randomisation. In recent years, the methodology to be employed in the present work has become standard in the assessment of the effects of health interventions (implementation research) [46].

### Ethical concerns

The described trial was evaluated and approved by the Central Committee of Primary Care Research in Madrid and the Clinical Research Ethics Committee of the Regional Community of Madrid. Information about the study, as well as its purpose and justification, will be provided to parents via the children’s school diaries. Parents/guardians will be asked to give their informed consent for their child to participate 10 days prior to the interventions; it will be collected by the class teacher from these same diaries. Consent is requested so that a child’s results can be assessed; no consent is required for the children to simply take part in the interventions since both fall within the official, free elective educational activities that can be chosen by the Madrid Region’s schools. A way for parents and class teachers to contact the principal investigator will be provided in order to resolve any queries that may arise.

## Abbreviations

AATRM: Catalan Agency for Technology and Medical Research; BMI: Body Mass Index; CDC: Centers for Disease Control and Prevention; CRF: Cardiovascular risk factors; DE: Design Effect; ICC: Intracluster Correlation Coefficient; IHD: Ischemic heart disease; IOT: International Obesity Taskforce; HT: Hypertension; NAOS: Strategy for Nutrition, Physical Activity and Obesity Prevention; NHANES: National Health and Nutrition Examination Survey; SIVFRENT: Surveillance System of Risk Factors associated with Non-Communicable Diseases; WHO: World Health Organization.

## Competing interests

The authors declare they have no competing interests.

## Authors’ contributions

BNA had the original idea for the study. BNA, FRS, LMSG, MJFL, MRL and AGG designed the protocol and wrote the drafts of the paper. MJFL, LMSG, LRD, RSA, BSG, AGG, PLB, SMV, MRL, DAP, JAP, CAC, AAV, JBA, ACS, AGA, JGP, IGP, MIM, PPC, ERC, ESA, CSF, GGJ, LVP and JLG participated in the writing and reading of the drafts. BNA is the author responsible for the study and for coordinating the research group. All the authors have read and approve the final manuscript.

## Authors’ information

The SAVINGHEARTS group is composed of: DANIEL ABAD PÉREZ, JUAN M. ALONSO PÉREZ, CELINA ARANA CAÑEDO-ARGUELLES, ANA B. ARRIBAS VELA, JUAN M BALLESTEROS ARRIBAS, ANA CUBILLO SERNA, MARÍA JESÚS FERNÁNDEZ LUQUE, ANGELA GALLEGO ARENAS, JULIA NATIVIDAD GARCIA PASCUAL, ILUMINADA GARCIA POLO, GEMA GIL JUBERIAS, AMELIA GONZÁLEZ GAMARRA, MARAVILLAS IZQUIERDO MARTÍNEZ, PILAR LOECHES BELINCHÓN, JAVIER LÓPEZ GONZÁLEZ, SOLEDAD MAYAYO VICENTE, BLANCA NOVELLA ARRIBAS, PILAR PÉREZ CAYUELA, FRANCISCO RODRÍGUEZ-SALVANÉS, ESTHER RUIZ CHÉRCOLES, LOURDES RUIZ DÍAZ, MARTA RUIZ LÓPEZ, ELENA SALVADOR ALCAIDE, ROSA SÁNCHEZ ALCALDE, LUIS MARÍA SÁNCHEZ GÓMEZ, BELÉN SIERRA GARCÍA, CARMEN SUAREZ FERNÁNDEZ AND LORENA VEGA PIRIS. Funding for the trial was provided by the *Plan Nacional de Investigación Científica, Desarrollo e Innovación Tecnológica (I+D+I)* under the auspices of the Instituto de Salud Carlos III – Fondo de Investigación Sanitaria, Expediente Nº PI11/00798.

## Pre-publication history

The pre-publication history for this paper can be accessed here:

http://www.biomedcentral.com/1471-2458/12/1024/prepub
